# Biallelic Truncating *DNAH14* Variant in Siblings with Neurodevelopmental Disorder and Predominant Ataxia: Clinical Report and Literature Review

**DOI:** 10.3390/ijms27020575

**Published:** 2026-01-06

**Authors:** Savas Baris, Mustafa Dogan, Kerem Terali, Alper Gezdirici, Recep Eroz, Peren Perk Yucel, Huseyin Kilic, Cuneyd Yavas, Gizem Yildirim, Ibrahim Baris

**Affiliations:** 1 Department of Medical Genetics, Aydın Maternity and Children’s Hospital, Aydin 09020, Turkey; brsbarsav@gmail.com; 2 Division of Genetics, Gelisim Medical Laboratories, Istanbul 34100, Turkey; 3 Department of Medical Biochemistry, Faculty of Medicine, Cyprus Health and Social Sciences University, Morphou 99750, Cyprus; kerem.terali@gmail.com; 4 Department of Medical Genetics, University of Health Sciences Basaksehir Cam and Sakura State Hospital, Istanbul 34480, Turkey; dralpergezdirici@gmail.com (A.G.); gizemmerve.y@gmail.com (G.Y.); 5 Department of Medical Genetics, Faculty of Medicine, Aksaray University, Aksaray 68100, Turkey; eroz38r@hotmail.com; 6 Department of Pediatric Neurology, Basaksehir Cam and Sakura State Hospital, Istanbul 34480, Turkey; perkperen@gmail.com; 7 Department of Pediatric Neurology, Cerrahpasa Faculty of Medicine, Istanbul University, Istanbul 34098, Turkey; kilichuseyin@me.com; 8 Department of Molecular Biology and Genetics, Biruni University, Istanbul 34015, Turkey; cyavas@biruni.edu.tr; 9 Department of Molecular Biology and Genetics, Koc University, Istanbul 34450, Turkey; ibaris@ku.edu.tr

**Keywords:** ataxia, neurodevelopment, bioinformatic, genetic disease, variant, *DNAH14*

## Abstract

Neurodevelopmental disorders (NDDs) with ataxia are genetically heterogeneous and remain a diagnostic challenge. Recent advances in genomic technologies have facilitated the identification of rare, potentially causative variants in genes not traditionally associated with classic NDD phenotypes. The *DNAH14* gene, encoding a dynein axonemal heavy chain involved in ciliary motility, has recently emerged as a novel candidate in neurological syndromes. Here, we report two Turkish siblings presenting with late-onset balance disorder, progressive ataxia, and cognitive impairment. Initial genetic analysis revealed that both siblings also harbor *FXN* GAA repeat expansions consistent with pathogenic Friedreich’s ataxia (FRDA). To elucidate the molecular basis of the patients’ cognitive impairment, whole-exome sequencing was performed. This analysis identified a novel homozygous frameshift variant in the *DNAH14* gene, located within the conserved linker domain upstream of the motor core, which is critical for ATP hydrolysis and microtubule interactions. The variant is absent from population databases, predicted to be deleterious by multiple in silico algorithms, and segregates in the family in a manner consistent with autosomal recessive inheritance. The coexistence of FRDA expansions and a truncating *DNAH14* variant suggests a potential dual genetic contribution to the observed phenotype, in which FRDA-associated pathology likely underlies the ataxia, while *DNAH14* disruption may contribute to additional neurodevelopmental features. This is the first report describing the co-occurrence of FRDA and a homozygous truncating *DNAH14* variant in the same individuals, broadening our understanding of overlapping neurogenetic mechanisms. Our findings expand the phenotypic spectrum of *DNAH14*-related disorders and highlight the importance of considering multilocus pathogenic variants in patients with complex or atypical ataxia presentations.

## 1. Introduction

Neurodevelopmental disorders (NDDs) are complex conditions encompassing a broad spectrum of neurological and developmental impairments, characterized by clinical and etiological heterogeneity. These disorders include global developmental delay and regression, intellectual disability, deficits in cognition, communication, behavior, or motor skills, microcephaly, epilepsy, and autism spectrum disorder [1,2]. Genetic factors play a significant role in the etiology of NDDs. In recent years, whole-exome sequencing (WES) has emerged as a powerful and scalable tool for identifying candidate genes, particularly in consanguineous populations. To date, more than 2500 genes associated with intellectual disability (ID), including primary and candidate genes, have been identified [3]. Advances in sequencing technologies now facilitate the rapid discovery of novel genes implicated in NDDs, shedding light on the heterogeneous genetic mechanisms underlying ID [4].

*DNAH14* (dynein axonemal heavy chain 14), located on chromosome 1q42, is a member of the axonemal dynein heavy chain family. These microtubule-associated motor proteins are essential for the function of motile cilia [5]. Axonemal dyneins generate force toward the minus ends of microtubules through ATPase activity, enabling ciliary and flagellar motion. While several axonemal dynein genes have been implicated in the etiology of primary ciliary dyskinesia (PCD) [6], the broader role of this protein family in human disease remains underexplored.

The *DNAH14* gene was first identified in 2015 as a candidate gene potentially linked to prenatal lethality [7]. Subsequent studies have associated *DNAH14* with diverse phenotypes, including panventriculomegaly, cerebral arteriovenous malformations, PCD, ID, and neurodevelopmental delay with seizures [8,9,10,11,12,13,14,15]. Despite these findings, the *DNAH14* gene has not yet been linked to a specific phenotype in the Online Mendelian Inheritance in Man (OMIM) database, and no large-scale studies have systematically evaluated its relationship with neurodevelopmental delay.

In this study, we present two siblings with neurodevelopmental delay and ataxia, carrying a homozygous pathogenic variant in *DNAH14* and pathogenic GAA repeat expansions in the FXN gene in both siblings, confirming a diagnosis of Friedreich’s ataxia (FRDA). FRDA, the most common hereditary ataxia, is characterized by progressive gait and limb ataxia, dysarthria, areflexia, and sensory neuropathy. Cognitive impairments in FRDA are typically mild, with subtle deficits in executive, fluency, and visuoconstructive domains [16]. The intellectual disability observed in our patients, therefore, exceeds the expected cognitive profile of FRDA and suggests additional contribution from *DNAH14* disruption.

## 2. Results

We report two siblings, a 22-year-old male (Proband 1, P1) and a 13-year-old male (Proband 2, P2), both presenting with late-onset balance disorders, frequent falls, ataxia, and impaired cognitive function. The siblings were born to consanguineous Turkish parents, with unremarkable prenatal, perinatal, and postnatal histories. Both pregnancies were uneventful, and there was no history of complications during delivery. The mother has had two pregnancies, with no history of miscarriage. Postnatal Apgar scores for the affected siblings are unavailable. Despite undergoing evaluations in multiple medical departments, a definitive diagnosis for the affected siblings had not been established prior to this study.

Neurological examinations revealed bilateral clumsiness during cerebellar tests and tandem gait in both siblings, who exhibited a widened support base while walking. Symptoms had been present for at least five years in the elder sibling (P1) and three years in the younger sibling (P2). Both patients exhibited intellectual disabilities that precluded attendance at regular schools. Their condition was not associated with regression, and clinical findings were non-progressive at least until their most recent follow-up. The family history revealed no similar cases, and clinical findings, along with the natural history of the affected siblings, are detailed in Table 1. For comparison, data on previously reported patients with biallelic variants in the *DNAH14* gene were also included. Notably, weight, height, and head circumference measurements of all three siblings were within the normal range, and no physical dysmorphic features were observed.

**Figure 1:**
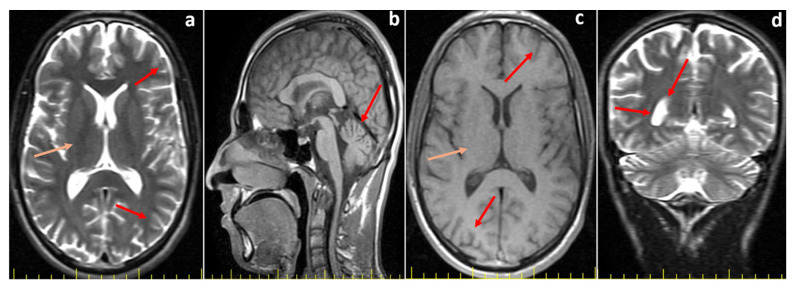
Cranial MR images of the index patient (Proband 1, P1) at 20 years of age. Cranial MR images of the index patient (Proband 1, P1) at 20 years of age. (**a**) T2-weighted axial image shows normal cerebral sulci (red arrows) and basal ganglia (orange arrow). (**b**) T1-weighted sagittal image reveals mild cerebellar atrophy (red arrow). (**c**) T1-weighted axial image shows normal cerebral sulci (red arrows) and basal ganglia (orange arrow). (**d**) T2-weighted coronal section demonstrates normal ventricular volume (red arrows).

**Figure 2:**
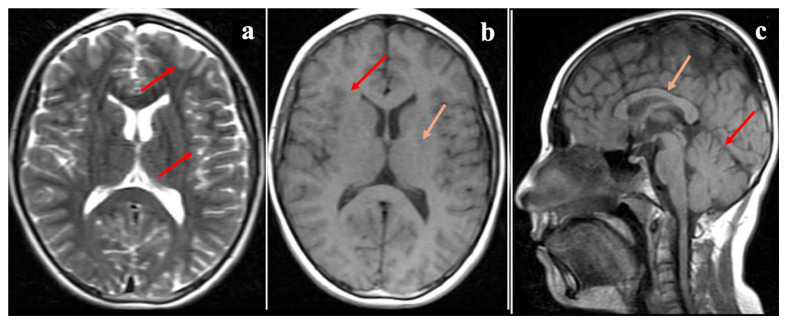
Cranial MR images of the younger sibling (Proband 2, P2) at 12 years of age. (**a**) T2-weighted axial image demonstrates normal cerebral sulci and gyri (red arrows). (**b**) T1-weighted axial image demonstrates normal cerebral sulci and gyri (red arrow) and basal ganglia (orange arrow). (**c**) T2-weighted axial image demonstrates a normal corpus callosum (orange arrow), with no evidence of cerebellar atrophy (red arrow).

Further neurological assessments revealed that both affected siblings demonstrated impaired cognitive function but were able to follow simple commands and communicate effectively. Muscle strength in the upper and lower extremities was preserved, sensory responses were normal, and deep tendon reflexes were hypoactive. There were no signs of extremity spasticity, pathological reflexes, or seizure activity. The Romberg test was positive, and both dysarthria and dysdiadochokinesia were present. Cranial MRI of the elder sibling (P1), performed at the age of 20, showed mild cerebellar atrophy (Figure 1), whereas the MRI of the younger sibling (P2), obtained at the age of 12, revealed no structural abnormalities (Figure 2). Eye movements were normal in both cases, and no tongue fasciculations or aphasia were observed. Additionally, plasma creatine kinase (CK) levels were within normal limits for both patients.

Electrophysiological evaluations were performed on the index patient (P1) using concentric needle electromyography (EMG) of the right upper and lower extremities. Sensory nerve studies revealed the absence of bilateral sural, superficial radial, and ulnar responses, along with reduced amplitudes in the bilateral superficial peroneal responses. The median nerve exhibited reduced response amplitude, prolonged distal latency, and slow conduction velocity on the right side. Motor nerve conduction studies were normal, and needle EMG revealed normal bioelectrical activity in the examined muscles. These findings, obtained under suboptimal cooperation conditions, were consistent with polyneuropathy affecting sensory fibers and mild carpal tunnel syndrome involving the sensory fibers of the right median nerve.

**Figure 3:**
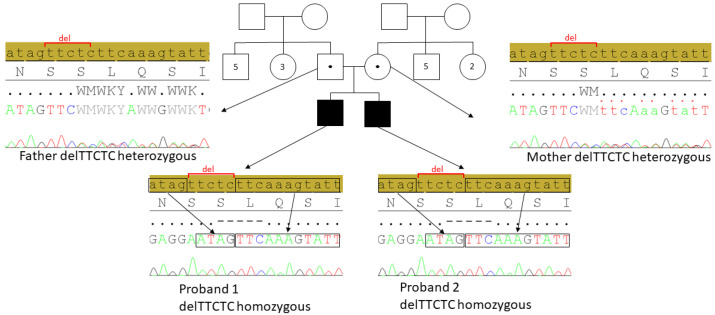
Pedigree of the family with *DNAH14*-related neurodevelopmental disorder. Sanger sequencing electropherograms confirm the presence of the c.3060_3064del variant in *DNAH14* in the homozygous state in both affected siblings (P1 and P2). The variant is present in the heterozygous state in both parents.

To investigate the etiology of ataxia, spinocerebellar ataxia panel testing and triplet repeat analysis for Friedreich ataxia were conducted. TP-PCR analysis revealed homozygous GAA expansion in Frataxin gene, confirming a diagnosis of FRDA [18]. Given the clinical presentation and imaging findings, a primary genetic neurological disorder was suspected, prompting WES, which identified a novel homozygous frameshift variant, NM_001367479.1: c.3060_3064del [p.Leu1021LysfsTer3], in the *DNAH14* gene. This variant was subsequently confirmed by Sanger sequencing and detected in a homozygous state in the younger affected sibling (P2), while both parents were found to be heterozygous carriers. Electropherogram views of the identified *DNAH14* variant in family members are shown in Figure 3. Notably, this variant is absent from public human genetic variant databases, including the Genome Aggregation Database (gnomAD) and ClinVar, and has not been previously reported in the scientific literature. Based on in silico predictions and familial segregation analysis, it is classified as pathogenic in accordance with the ACMG/AMP guidelines for variant interpretation [19].

**Figure 4:**
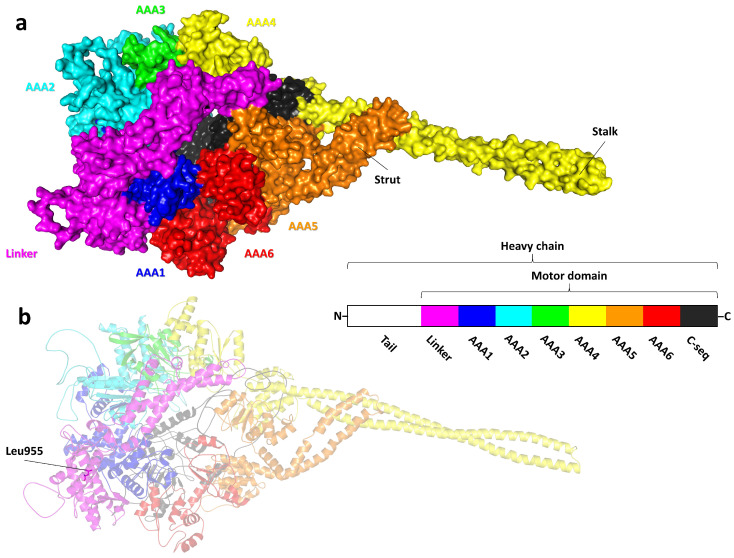
Overall structure of the axonemal dynein motor domain. (**a**) Surface representation of a homology model of the motor domain of human wild-type dynein axonemal heavy chain 14, with individual subdomains color-coded. (**b**) Transparent ribbon representation of the same model, showing the affected amino acid residue (Leu955) in stick representation. Inset: schematic of the domain architecture, using the same color scheme.

In an attempt to visualize the potential impact of the identified gene variant on the corresponding protein’s conformation, stability, and function, we used a comparative model of human dynein axonemal heavy chain 14 (Figure 4). This model was based on a 2.8 Å x-ray structure of the *Dictyostelium discoideum* dynein motor domain, which lacks the microtubule-binding subdomain (PDB ID: 3VKG) [20] and is archived in ModBase (Accessed Date: http://salilab.org/modbase; accessed on 10 November 2025). As inferred from protein homology, the domain organization of human dynein axonemal heavy chain 14 follows the canonical dynein heavy chain structure, featuring specialized adaptations for its role in axonemal function. Similar to other dynein heavy chains, human dynein axonemal heavy chain 14 consists of an N-terminal tail domain and a motor domain. The tail domain plays a critical role in binding accessory proteins and regulatory subunits, thereby facilitating the assembly of the dynein complex and ensuring proper ciliary localization. The motor domain is further subdivided into a linker subdomain, six AAA+ (ATPase associated with diverse cellular activities) modules (AAA1–AAA6), a coiled-coil stalk and strut, and a C-terminal sequence, which together drive the motor’s ATP-dependent activity and ciliary motility. At the tip of the coiled-coil stalk, which protrudes from AAA4, resides a microtubule-binding subdomain (missing in the present protein model) essential for dynein’s interaction with microtubules.

It is worth noting that Leu1021 in the isoform encoded by the mRNA variant NM_001367479.1 (UniProtKB ID: A0A804HLD3; 4617 residues) corresponds to Leu955 in the canonical sequence (UniProtKB ID: Q0VDD8; 3507 residues) upon which the comparative protein model was based. The p.Leu955LysfsTer3 variant introduces a frameshift at position 955, replacing leucine with lysine, followed by two missense residues before a premature stop codon at position 958. This variation produces a truncated protein of 957 amino acids, preserving only approximately 27% of the native 3507-residue protein. Critically, this frameshift truncates the protein within the linker subdomain, leading to the complete loss of essential downstream elements, including the coiled-coil stalk and strut, the microtubule-binding subdomain, and the six AAA+ modules, which are indispensable for ATP hydrolysis and microtubule-based motility. As a result, the protein is rendered entirely non-functional as an axonemal dynein heavy chain. While theoretical considerations might suggest that such a truncated protein could interfere with the assembly or function of axonemal dynein complexes, a dominant-negative effect is unlikely in this context, as both parents of the affected individuals are healthy heterozygotes. This supports a loss-of-function mechanism consistent with autosomal recessive inheritance. Alternatively, the mutant transcript may be targeted for degradation via nonsense-mediated decay, depending on its position relative to the final exon–exon junction, which would further contribute to the absence of functional protein.

## 3. Discussion

Comprehensive genetic analysis revealed that both siblings harbored pathogenic GAA repeat expansions in the FXN gene, and a novel homozygous c.3060_3064del (p.Leu1021LysfsTer3) variation in the *DNAH14* gene. This dual molecular finding provides a more comprehensive explanation for their phenotype: the ataxia and cerebellar features align with FRDA pathology, while the coexisting intellectual disability is atypical for FRDA and may reflect the additive effect of the *DNAH14* truncating variant.

Several axonemal dynein genes have been implicated in the etiology of primary ciliary dyskinesia [6]; however, *DNAH14* has not yet been associated with a documented phenotype in the OMIM database. According to the BioGPS database (http://biogps.org; accessed on 10 November 2025), *DNAH14* is widely expressed across human tissues. In the central nervous system, its protein is predominantly localized to ependymal cells and choroid plexus epithelial cells, which are rich in motile cilia [15]. These ciliated structures play a crucial role in cerebrospinal fluid (CSF) flow, suggesting a functional significance for *DNAH14* in maintaining normal neurophysiological processes.

Developmentally, *DNAH14* exhibits a temporal expression pattern. Data from BrainSpan (http://www.brainspan.org/; accessed on 10 November 2025) [21] indicate higher expression levels during embryonic stages, with a notable decline postnatally. This pattern suggests that *DNAH14* may play a critical role in embryonic neurodevelopment, particularly in motile cilia activity. Additionally, it has been proposed that *DNAH14* might have either a direct or indirect impact on neurodevelopmental processes [11]. Recent studies have linked *DNAH14* variants to a spectrum of neurodevelopmental disorders, including global developmental delay, seizures, microcephaly, and hypotonia [10,11,12,13].

Dysfunction of motile cilia in ependymal cells has been proposed as a pathogenic cause of excessive CSF accumulation, leading to ventricular enlargement in hydrocephalus [22,23]. It has been hypothesized that certain cases of hydrocephalus associated with ciliary gene mutations may not only result from the loss of cilia-driven CSF flow but also from altered neurodevelopment, considering the potential roles of ciliary genes in signaling and neural stem cell fate determination [24,25,26]. Cytoplasmic dynein motor protein classes are known to be involved in interkinetic nuclear migration of ventricular neural stem cells and retrograde axonal transport during neurogenesis [27].

**Figure 5:**
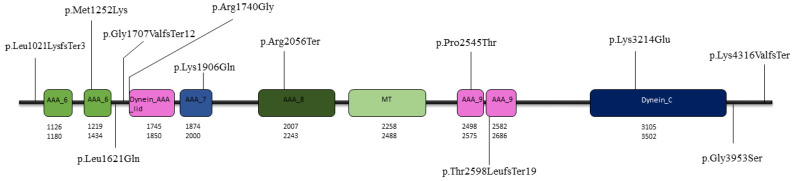
Schematic representation of the domain architecture of the 3507 amino acid DNAH14 protein and distribution of the identified variants. The dynein heavy chain AAA lid domain (Dynein_AAA_lid), P-loop–containing dynein motor region (AAA_7), P-loop–containing dynein motor region D4 (AAA_8), ATP-binding dynein motor region (AAA_9), microtubule-binding stalk of the dynein motor (MT), and the dynein heavy chain C-terminal domain (Dynein_C) are indicated by colored boxes. Missense, nonsense, and frameshift variants are mapped along the protein according to their amino acid positions.

Comprehensive analysis of all published patients with biallelic *DNAH14* variants reveals phenotypic heterogenity rather than a single clinical entity. As of December 2025, 13 disease-causing variants were reported in Pubmed (Figure 5). Across all reported cases, biallelic *DNAH14* variants give rise to a broad clinical spectrum ranging from PCD to neurodevelopmental disorders. At present, only limited genotype–phenotype correlations can be proposed for *DNAH14*, and these should be interpreted with caution. Review of all reported *DNAH14* variants to date, including our cases, are summarized in Table 1. The DNAH14 protein is large and functionally complex, comprising multiple conserved domains including linker regions, AAA+ ATPase domains, and microtubule-binding regions. Pathogenic variants reported so far are distributed throughout the gene and include frameshift, nonsense, missense, and splice-region variants. Importantly, similar clinical phenotypes have been observed in patients with variants affecting different regions of the protein, while conversely, distinct phenotypes have been reported for variants located within comparable domains. For example, truncating variants located in the N-terminal and central regions of the protein, including the frameshift variant identified in our patients (p.Leu1021LysfsTer3), are associated with neurodevelopmental phenotypes characterized by ataxia, cognitive impairment, or intellectual disability. However, truncating or missense variants affecting more distal regions of the protein have been linked to a broader clinical spectrum, including primary ciliary dyskinesia-like respiratory features, autism spectrum disorder, or multisystem involvement. This variability argues against a simple linear relationship between mutation position and clinical severity.

In 2015, Shamseldin et al. conducted a WES analysis to identify monogenic causes of embryonic lethality in humans [7]. The study included 24 consanguineous families with multiple pregnancies diagnosed with lethal non-immune hydrops fetalis. In one family, a homozygous variant in *DNAH14* was detected. However, the authors emphasized that, at the time, there was insufficient evidence to establish a direct link between *DNAH14* variants and embryonic lethality, as the gene had not been previously associated with human diseases. Nevertheless, *DNAH14* was reported as a candidate gene potentially contributing to embryonic lethality.

In 2016, Kageyama et al. analyzed patients with panventriculomegaly (PaVM), a unique form of hydrocephalus characterized by an enlarged foramen of Magendie and a prominent cisterna magna [15]. Using copy number variation analysis, they identified *DNAH14* as a possible causative gene in a Japanese family with three affected individuals. A 322 kb heterozygous deletion at 1q42.12 involving *DNAH14* was detected. Since *DNAH14* encodes an axonemal dynein in motile cilia, the authors suggested that its deletion may disrupt cilia function during hydrocephalus pathogenesis. Additionally, two of the three affected patients exhibited cognitive impairment, while the third had gait disturbances. These findings suggest that next-generation sequencing (NGS) may identify additional *DNAH14* variants in patients with hydrocephalus-associated mutations.

In 2020, Zhang et al. performed trio exome sequencing to investigate the molecular etiology of brain arteriovenous malformations (BAVMs) [14]. They identified compound heterozygous variants in *DNAH14* in two unrelated families. Although there was no direct evidence linking *DNAH14* to angiogenesis or other vascular disorders, their in silico analyses and segregation studies suggested that *DNAH14* could be involved in BAVM pathogenesis.

A 2017 poster presentation by Macaya et al. explored the utility of WES in patients with nonprogressive congenital ataxia [17]. Among 13 patients, a homozygous variant in *DNAH14* was identified, suggesting *DNAH14* as a novel candidate gene for this phenotype. All patients presented with hypotonia and developmental delay in infancy, followed by ataxia. Notably, intellectual disability was reported in 10 out of 13 patients, but there were no significant familial findings.

In 2018, Al-Nabhani et al. reanalyzed exome sequencing data from patients with ID and reported a homozygous missense variant in *DNAH14* [13]. They suggested *DNAH14* as a candidate gene for ID in a male patient with global developmental delay, hypotonia, and neuroregression, despite a normal cranial MRI and metabolic screening. Similarly, Al-Kasbi et al. (2022) identified a homozygous significant variant in *DNAH14* in a 7-year-old male patient in a study of Middle Eastern families with ID [12].

In 2022, Li et al. performed trio WES and identified three unrelated patients with compound heterozygous *DNAH14* variants [11]. These patients exhibited early-onset EEG abnormalities, along with seizures, neuromotor and developmental delay, hypotonia, and microcephaly. Detailed clinical and genetic data for these patients are summarized in Table 1.

Trost et al. (2022) conducted a whole-genome sequencing (WGS) study to identify the genetic etiology of autism spectrum disorder (ASD) [10]. They detected a homozygous mutation in *DNAH14* in four unrelated patients. While detailed clinical data were not available, all individuals met the DSM-5 criteria for ASD (American Psychiatric Association, 2020, https://www.psychiatry.org/Psychiatrists/Practice/DSM/Updates-to-DSM/Coding-Updates/2020-Coding-Updates; accessed on 10 November 2025).

In 2022, Duy et al. analyzed a published single-cell RNA sequencing dataset of the developing human brain [28] and identified strong expression of *DNAH14* in neuroepithelial and radial glial progenitors [26]. This suggests that *DNAH14* may have a neurodevelopmental role beyond its established function in motile cilia. Reports of *DNAH14*-related cases in the literature, describing patients with microcephaly, hypotonia, seizures, ID, and ataxia, further support this hypothesis. Consistent with the idea that dynein mutations may affect neurodevelopment in addition to fluid dynamics, mutations in the dynein gene *DNAH2* have been previously reported in patients with primary microcephaly [29].

In 2021, Guan et al. investigated the clinical and genetic spectrum of PCD in Chinese children [9]. They identified compound heterozygous *DNAH14* variants in a 5-year-old male patient presenting with recurrent wet cough, bronchiectasis, chronic sinusitis, and reduced nasal nitric oxide (nNO) levels (63 nL/min). Transmission electron microscopy (TEM) showed shortened or truncated outer dynein arms (ODAs). Immunofluorescence staining revealed reduced *DNAH14* expression along the ciliary axoneme in respiratory epithelial cells compared to healthy controls. Trio exome sequencing confirmed the segregation of *DNAH14* variants within the family, supporting a definitive PCD diagnosis. More recently, Jat et al. (2024) reported another PCD case in India involving an 8-year-old girl with persistent wet cough and unexplained neonatal respiratory distress [8]. No situs abnormalities were detected. Her nNO level was 31.8 nL/min, and high-speed video microscopy revealed immobile cilia. TEM analysis demonstrated the absence of both inner (IDA) and outer dynein arms (ODA), further supporting a *DNAH14*-related PCD diagnosis.

PCD is a rare, genetically heterogeneous ciliopathy characterized by neonatal respiratory distress, chronic wet cough, sinusitis, bronchiectasis, otitis media, infertility, and situs inversus in approximately 50% of affected individuals [6]. While the estimated prevalence of heterotaxy in the general population is approximately 1 in 10,000 pregnancies, recent studies indicate that about 50% of PCD patients have organ laterality defects, and nearly 6% present with cardiovascular malformations [30,31]. Although more than 50 genes involved in ciliary structure and function have been linked to the PCD phenotype, a genetic cause remains unidentified in 20–30% of individuals with a definitive PCD diagnosis [32]. In the literature, *DNAH14* homozygous mutations have been associated with PCD in two independent families across two separate studies [8,9]. The identification of new PCD-associated genes will not only facilitate disease diagnosis but also contribute to the development of therapeutic interventions and enhance our understanding of PCD pathogenesis.

Approximately 50% of PCD patients exhibit left–right laterality defects due to dysfunction of embryonic nodal cilia [33]. However, in our study evaluating patients with mutations in *DNAH14*, no cases of laterality defects were reported. After first identifying *DNAH14* as a new PCD-related gene in 2021 [9], Li and colleagues later detected compound heterozygous variants in *DNAH14* in three independent patients with NDD in 2022 [11]. Notably, two of these three patients had a history of pneumonia, leading Li and colleagues to suggest that *DNAH14* variants may increase susceptibility to respiratory infections. Although recurrent respiratory problems were not reported in our patients or in other *DNAH14*-related NDD families described in the literature, we suggest that this potential association warrants further investigation.

The possible link between *DNAH14* and the neurodevelopmental symptoms in the patients is hypothesized as follows: *DNAH14* encodes an axonemal dynein heavy chain that is predominantly expressed in ependymal cells lining the ventricular system and in epithelial cells of the choroid plexus, both of which are key components of the brain–CSF interface. Ependymal cells are multiciliated cells whose coordinated ciliary beating contributes to local CSF flow along the ventricular walls, while the choroid plexus regulates CSF production and composition [24,26]. Crucial steps of cortical neurogenesis occur at the brain–cerebrospinal fluid (CSF) interface, where neural stem cells (NSCs) undergo neurogenic divisions along the ventricular wall, giving rise to progenitor cells that radially migrate toward the developing cortical plate [34]. Ependymal cells arise from ventricular neural stem cells during late gestation and early postnatal life, establishing a developmental link between embryonic neurogenesis and postnatal ventricular function [35,36]. Consequently, defects in proteins enriched in mature ependymal cells may reflect earlier perturbations in neural progenitor biology rather than isolated postnatal dysfunction. In support of this model, recent human genetic studies of congenital hydrocephalus and related neurodevelopmental disorders demonstrate that many disease-associated genes (*TRIM71*, *SMARCC1*/*SMARCA4*, *PTEN*, *KAT6B*, *SETD2*, *CHD7*, *DYNC1H1*, *KIF2A*, *LIS1*) are highly expressed in ventricular zone neural stem cells (NSCs) and radial glial progenitors during prenatal brain development, rather than genes specific to mature ependymal cells [24,26,37]. Consistent with this statement, *DNAH14* gene expression is high during the embryonic stage and declines after birth based on BrainSpan (http://www.brainspan.org/; accessed on 10 November 2025).

While the presence of GAA expansions in *FXN* establishes Friedreich’s ataxia as a major contributor to the ataxic presentation, the coexistence of a homozygous truncating *DNAH14* variant likely modifies the clinical phenotype. In classic FRDA, cognitive involvement is subtle, primarily affecting executive and visuoconstructive functions, with no evidence of global intellectual disability [16]. Therefore, the intellectual disability in our patients cannot be solely attributed to FRDA, supporting a dual molecular diagnosis. This observation underscores the importance of multilocus pathogenicity in neurogenetic disorders, where concurrent mutations in distinct genes may act synergistically to produce atypical or more severe phenotypes. The current findings broaden the phenotypic spectrum of *DNAH14*-related disorders and highlight its potential role in neurodevelopmental processes beyond ciliary function.

Induced pluripotent stem cells (iPSCs) provide a powerful tool to investigate human disease mechanisms [38]. iPSCs can be derived directly from patients carrying pathogenic *DNAH14* variants and differentiated into relevant neural cell types. For example, iPSCs can be differentiated into region-specific neuronal subtypes (such as cortical, cerebellar, or sensory neurons) as well as glial cells, allowing evaluation of *DNAH14* function in post-mitotic neural populations. Dynein-mediated axonal transport is essential for neuronal survival, synaptic maintenance, and mitochondrial trafficking, and iPSC-derived neurons can be used to determine whether *DNAH14* dysfunction leads to impaired intracellular transport, mitochondrial mislocalization, synaptic abnormalities, or increased vulnerability to cellular stress—features commonly shared by neurodegenerative disorders. Furthermore, differentiation of iPSCs into ependymal-like cells and choroid plexus epithelial cells offers a unique opportunity to study *DNAH14* in cell types in which it is highly expressed, enabling investigation of ciliary structure, motility, and signaling, as well as potential non-ciliary roles in intracellular transport and cell polarity. Importantly, iPSC-based ventricular niche models can be used to explore how alterations at the brain–CSF interface influence neuronal health over time, including changes in trophic factor secretion, inflammatory signaling, or metabolic support, all of which are increasingly implicated in neurodegenerative processes. Finally, integration of iPSC-derived models with transcriptomic, proteomic, and live-cell imaging approaches provides a comprehensive framework to identify molecular pathways underlying *DNAH14*-associated pathogenesis and to link early cellular dysfunction with progressive neurological disease.

## 4. Methods and Materials

Informed consent for medical examinations, genomic analyses, and case presentations was obtained from the patients and their parents. All procedures adhered to the principles of the Declaration of Helsinki and were approved by the institutional review board (Ethical Approval Number: 2025-07, Approval Date: 8 January 2025). A comprehensive clinical history was obtained, and neurological examinations were performed for all family members.

Genomic DNA was extracted from peripheral leukocytes of fresh blood samples collected from the index patient, and FRDA expansion was investigated by TP PCR [18]. Array comparative genomic hybridization (aCGH) was performed using the CytoScan Optima Assay (Affymetrix, Thermo Fisher Scientific, Waltham, MA, USA). As no genetic anomalies were identified through these tests, WES was conducted. The coding regions and splice sites of targeted genes were captured using the Illumina SureSelect V6 Exome kit. After library enrichment and quality control, sequencing was carried out on an Illumina HiSeq4000 platform, generating 100 bp paired-end reads at an average sequencing depth of 100×.

Raw reads were quality-trimmed using Trimmomatic (v0.39), and high-quality reads were mapped to the reference human genome (hg19) using the Burrows-Wheeler Alignment Tool (BWA). Duplicate reads were marked using Picard, a command-line Java tool for processing SAM and BAM files. Variant calling was performed using the Genome Analysis Toolkit (GATK) software package (v4). The following GATK modules were applied sequentially: RealignerTargetCreator, IndelRealigner, BaseRecalibrator, PrintReads, HaplotypeCaller, SelectVariants, VariantFiltration, and CombineVariants, to identify and filter single nucleotide polymorphisms (SNPs) and indels.

The detected variants were annotated using multiple databases and tools, including Illumina BaseSpace Variant Interpreter, InterVar, Franklin, VarSome, ClinVar, OMIM, and PubMed. Variants with a population frequency higher than 0.5% were excluded. The dbNSFP database, which integrates predictive tools such as SIFT, PolyPhen-2, LRT, and MutationTaster, was utilized to assess the pathogenicity and deleterious potential of variants.

Rare variants were classified according to the guidelines established by the American College of Medical Genetics and Genomics and the Association for Molecular Pathology (ACMG/AMP) for the interpretation of sequence variants [19]. Confirmation of the identified variant within the family was performed using Sanger sequencing on an ABI PRISM 3130 Genetic Analyzer (Applied Biosystems, Thermo Fisher Scientific, Tokyo, Japan).

For this study, 18 articles related to the *DNAH14* gene available in the PubMed database were reviewed. The main findings from the previously reported cases in the literature, along with those of the present index cases, are summarized in Table 1.

## 5. Conclusions

Recent studies suggest a broad spectrum of phenotypes associated with *DNAH14* mutations, ranging from neurodevelopmental disorders to ciliopathies. While its role in axonemal motility is well-established, its involvement in brain development and function remains an intriguing avenue for further research. *DNAH14* encodes a vital component of the axonemal dynein complex, with widespread expression and specific localization to ciliated cells, underscoring its importance in physiological processes, particularly during embryonic neurodevelopment. However, the role of motile cilia in neurodevelopment and brain function has not been fully elucidated. Additional studies are needed to explore the detailed molecular mechanisms underlying *DNAH14*-associated neurodevelopmental disease pathogenesis. Another limitation is the lack of and limited clinical data in our retrospective systematic review, which constrains a comprehensive assessment of the phenotype. Further validation of genotype–phenotype associations from recently published studies is necessary to enhance our understanding of the clinical implications of *DNAH14* mutations. Future studies utilizing NGS, functional assays, and model organisms will be critical in elucidating the underlying mechanisms in *DNAH14*-related disorders and supporting improved diagnosis and therapeutic strategies.

## Figures and Tables

**Table 1:** Clinical findings and natural history of the *DNAH14*-related neurodevelopmental disorder in patients known to date.

Report	Present Study P1	Present Study P2	Shamseldin et al. [7]	Macaya et al. [17]	Al-Nabhani et al. [13]	Zhang et al. [14]	Guan et al. [9]	Al-Kasbi et al. [12]	Li et al. [11]	Li et al. [11]	Li et al. [11]	Trost et al. [10]	Jat et al. [8]
Sex	Male	Male	NA	NA	Male	NA	Male	Male	Female	Female	Male	NA	Female
Origin	Turkish	Turkish	Arab	NA	Omani	Chinese	Chinese	Omani	Chinese	Chinese	Chinese	NA	Indian
Age at last exam (years)	22	13	-	-	-	NA	5	7	2	10	3	NA	8
Gestation	Term	Term	In utero abortus	Term	NA	-	Term	-	Term	Term	Term	NA	Term
Perinatal complication	-	-	Hydrops fetalis	-	NA	-	-	NA	-	-	-	NA	NA
Developmental delay	-	-	-	+	+	-	-	-	+	-	+	+	-
Intellectual disability	+	+		+	+	-	-	+	+	-	-	+	-
Motor delay	-	-	-	+	+	-	-	-	+	-	+	+	-
Speech delay	-	-	-	-	-	-	-	-	+	-	+	+	-
Cognitive delay	+	+	-	+	+	-	-	+	+	-	+	+	-
Microcephaly	-	-	-	-	-	-	-	-	-	-	+	NA	-
Hypotonia	-	-	-	+	+	-	-	-	+	-	+	NA	-
Seizure history	-	-	-	-	-	-	-	-	+	+	+	NA	-
Pneumonia	-	-	-	-	-	-	+	-	+	-	+	NA	+
Other findings	Ataxia, late-onset balance disorder	Ataxia, late-onset balance disorder	Embryonic lethality, habitual abortus	Ataxia, normal metabolism	Neuro-regression, normal metabolism	Non-syndromic BAVM	Recurrent wet cough, bronchiectasis, chronic sinusitis	A brother with similar findings	-	Difficulty in swallowing	M, SS, PFO	7 unrelated patients with ASD phenotype	Persistent wet cough, neonatal RDS, no situs abnormality
Variants (NM_001367479)	c.3060_3064del	c.3060_3064del	c.3755T > A	NA	c.9640A > G	NA	c.5120del c.4255-22_4255-21dup	c.5716A>C	c.6166C > T c.5218A > G	c.12946_12947del c.4862T > A	c.7633C > A c.11857G > A	NA	c.7792del
Amino acid change	p.Leu1021LysfsTer3	p.Leu1021LysfsTer3	p.Met1252Lys	NA	p.Lys3214Glu	NA	p.Gly1707ValfsTer12	p.Lys1906Gln	p.Arg2056Ter p.Arg1740Gly	p.Lys4316ValfsTer p.Leu1621Gln	p.Pro2545Thr p.Gly3953Ser	NA	p.Thr2598LeufsTer19
Novelty	Novel	Novel	rs786205668	NA	rs547435453	NA	rs577893006 rs77953334	rs868380191	rs1394394477 rs190061594	rs1490181827 rs1271973468	NA rs1176041827		NA
Variant Zygosity	HM	HM	HM	HM	HM	CH	CH	HM	CH	CH	CH	NA	HM
Type of mutation	Frameshift	Frameshift	Missense	NA	Missense	NA	Frameshift Frameshift	Missense	Nonsense/ Missense	Frameshift/ Missense	Missense/ Missense	NA	Frameshift
Cranial MRI	Mild CA	-	-	NA	Normal	BAVM	-	NA	DWM, CCA	-	-	NA	-

P: patient; BAVM: brain arteriovenous malformation; DWM: decreased white matter; ASD: autism spectrum disorder; CCA: corpus callosum agenesis; CA: cerebral atrophy; HM: homozygous; CH: compound heterozygous; NA: not available; M: malnutrition; SS: short stature; PFO: patent foramen ovale; RDS: respiratory distress syndrome; +: presence of phenotype; -: absence of phenotype.

## Data Availability

The original contributions presented in this study are included in the article material. Further inquiries can be directed to the corresponding author.
